# Pelvic Organ Prolapse Reconstruction with the Chitosan-Based Novel Haemostatic Agent in Ovine Model—Preliminary Report

**DOI:** 10.3390/ijms25073801

**Published:** 2024-03-28

**Authors:** Klaudia Stangel-Wójcikiewicz, Maciej Murawski, Tomasz Schwarz, Krzysztof Skotniczny, Agnieszka Fuchs, Jan Wolski, Julia Radwan-Pragłowska, Łukasz Janus, Marek Piątkowski, Marta Kot, Andrzej Wróbel, Dorota Wojtysiak, Przemysław Urbaniec

**Affiliations:** 1 Department of Gynecology and Oncology, Faculty of Medicine, Jagiellonian University Medical College, ul. Kopernika 23, 31-501 Kraków, Poland; klaudia.stangel-wojcikiewicz@uj.edu.pl (K.S.-W.); krzysztof.skotniczny@uj.edu.pl (K.S.); 2 Department of Animal Nutrition, Biotechnology and Fisheries, Faculty of Animal Science, University of Agriculture in Kraków, ul. Mickiewicza 21, 31-120 Kraków, Poland; rzmmuraw@cyf-kr.edu.pl; 3 Department of Genetics, Animal Breeding and Ethology, Faculty of Animal Science, University of Agriculture in Kraków, ul. Mickiewicza 21, 31-120 Kraków, Poland; tomasz.schwarz@urk.edu.pl (T.S.); wojtysiakd@wp.pl (D.W.); 4 Department of Gynecological Endocrinology and Gynecology, Faculty of Medicine, Jagiellonian University Medical College, ul. Kopernika 23, 31-501 Kraków, Poland; konfgin@op.pl (A.F.); jasiekwolski@gmail.com (J.W.); 5 Department of Biotechnology and Physical Chemistry, Faculty of Chemical Engineering and Technology, Cracow University of Technology, ul. Warszawska 24, 31-155 Kraków, Poland; krakchemia@gmail.com (Ł.J.); marek.piatkowski@pk.edu.pl (M.P.); 6 Department of Transplantation, Institute of Pediatrics, Faculty of Medicine, Jagiellonian University Medical College, ul. Wielicka 265, 30-663 Kraków, Poland; marta.kot@uj.edu.pl; 7 Second Department of Gynecology, Medical University of Lublin, ul. Jaczewskiego 8, 20-090 Lublin, Poland; wrobelandrzej@yahoo.com; 8 The University Hospital in Kraków, ul, Kopernika 36, 31-501 Kraków, Poland; przemek.urbaniec@gmail.com

**Keywords:** pelvic organ prolapse, biomaterials, animal model, chitosan haemostatic agent, ovine

## Abstract

This prospective study aimed to assess the feasibility of chitosan biomaterial and subcutaneous gel implantation in an ovine model, with implications for women with genital prolapse. Twenty-four ewes were divided into four groups (n = 6 per group): chitosan type B, chitosan type C, chitosan unmodified injections, and polypropylene mesh. Ovine models were chosen due to their morphological resemblance to human reproductive organs. Animals were sacrificed after 90 days for macroscopic, pathomorphological, and immunohistochemical analysis. In the chitosan type B group, IL-6 and IL-10 levels decreased after 28 days, while chitosan type C and injection groups exhibited higher IL-6 than IL-10 levels. The polypropylene group displayed the highest IL-6 and lowest IL-10 levels. Histological examination of the polypropylene group revealed no degenerative changes or inflammation, whereas chitosan injection induced local inflammation. Other groups exhibited no degenerative changes. Ewes implanted with chitosan displayed reduced inflammation compared to polypropylene-implanted ewes. Chitosan implantation facilitated vaginal tissue healing, in contrast to polypropylene mesh, which led to extrusion. While chitosan holds promise as an alternative to polypropylene mesh, further research is imperative for comprehensive evaluation. This study suggests the potential of a chitosan biomaterial in pelvic organ prolapse treatment, warranting additional investigation.

## 1. Introduction

Our study includes immunohistochemical and histopathological assessments, and the assessments of the concentration of interleukins after implantation of the chitosan material into the vaginal wall of an ovine model.

In this investigation, we evaluated a chitosan biomaterial implanted into the anterior vaginal wall of a sheep model. The goal was to conduct a clinical evaluation of its viability for urogynecological disorders through surgical interventions. Our study findings were derived from a multifaceted analysis, encompassing histopathological assessments, immunohistochemical investigations, and the quantification of IL-6 and IL-10 levels across varying time points within the experiment. This approach enabled a thorough assessment of the inflammatory response and formed the basis for our study’s conclusions.

The global interest in tissue engineering and scaffold-based approaches arises from the inherent limitations of current therapeutic modalities such as tissue transplantation and artificial graft implantation. These strategies, aimed at restoring compromised anatomical structures, remain crucial in the surgical management of female genital prolapse—an affliction affecting over 50% of menopausal women. This condition leads to urogynecological disorders, urinary and fecal incontinence, pain, urogenital inflammation, and dyspareunia [1,2,3]. However, the utilization of polypropylene materials, the most prevalent choice until now, can induce complications like vaginal, bladder, or rectal extrusion, as well as erosion [4].

The USA FDA advisories issued in 2008 and 2011 regarding these materials have expanded scientific exploration toward an alternative, biodegradable, and non-toxic surgical substrate. In the realm of tissue engineering, the logical progression involves personalized implants derived from a patient’s own cells, enabling enhanced therapeutic response and efficacy. The combination of advance materials and cells holds the potential to yield constructs that promote tissue regeneration—a progression marked by mechanical resilience before final modulations in elasticity.

Emerging as an intriguing prospect, chitosan materials sourced from shellfish exhibit unique anti-inflammatory attributes and navigate a pre-scheduled biodegradation process, ensuring compatibility with surrounding tissues [5]. Natural polymers have seized attention due to their biocompatibility and affinity with extracellular matrix components. Consequently, natural polymers, endowed with biological activity and widespread availability, have emerged as a focal point for the development of novel natural or semi-synthetic materials. Notably, alginate, hyaluronic acid, starch, cellulose, collagen, chitin, and chitosan have garnered attention as enticing natural polymers conducive to tissue regeneration [6]. Among these, chitosan holds particular significance, boasting commendable properties such as high biocompatibility, biodegradability, antibacterial prowess, non-antigenicity, and potent adsorption capacities, rendering it an auspicious contender for tissue engineering and diverse biomedical applications [6]. Moreover, this biopolymer exhibits hemostatic activity, which significantly increases its usefulness during clinical procedures since it prevents undesired hemorrhages and massive blood loss, as uncontrolled bleeding during surgery can be associated with multiple post-trauma effects [7].

The objective of our investigation centered on investigating the immunological and pathomorphological responses in an animal model—a ewe subjected to hemostatic chitosan biomaterial and subcutaneous gel implantation. Additionally, our inquiry aimed to ascertain the feasibility of such implantation strategies for addressing genital prolapse in women.

## 2. Results

### 2.1. Chitosan Biomaterials: Characterization

**Figure 1:**
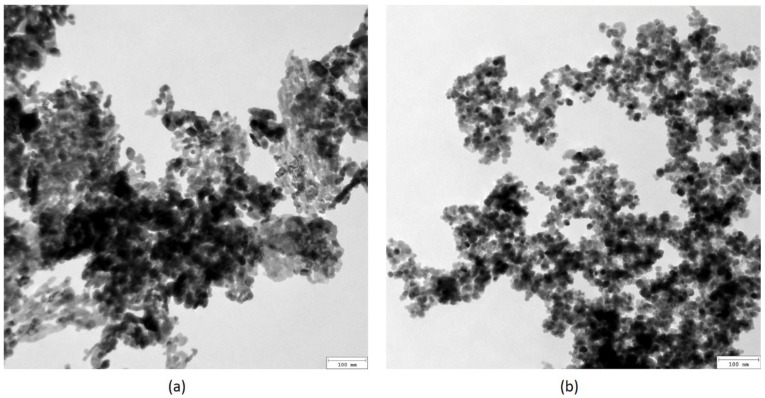
TEM microphotographs of the nanoparticles: (**a**) ZnO; (**b**) Fe_3_O_4_.

Figure 1 presents TEM microphotographs of the nanoparticles used for biomaterials’ preparation. ZnO nanoparticles (Figure 1a) are characterized by their baguette-like shape. No sharp edges are present. The NPs are of uniform size distribution, with average lengths of approximately 20 nm and diameters of 10 nm. Fe_3_O_4_ NPs, on the other hand, are of a circular, regular shape, with an average diameter of 20 nm. Such parameters are favorable for biomedical applications since they prevent bioaccumulation in the kidneys or liver. Additionally, they should not cause cell membrane damage or induce reactive oxygen species generation. In both cases, no residues or potentially cytotoxic contaminants were present.

**Figure 2:**
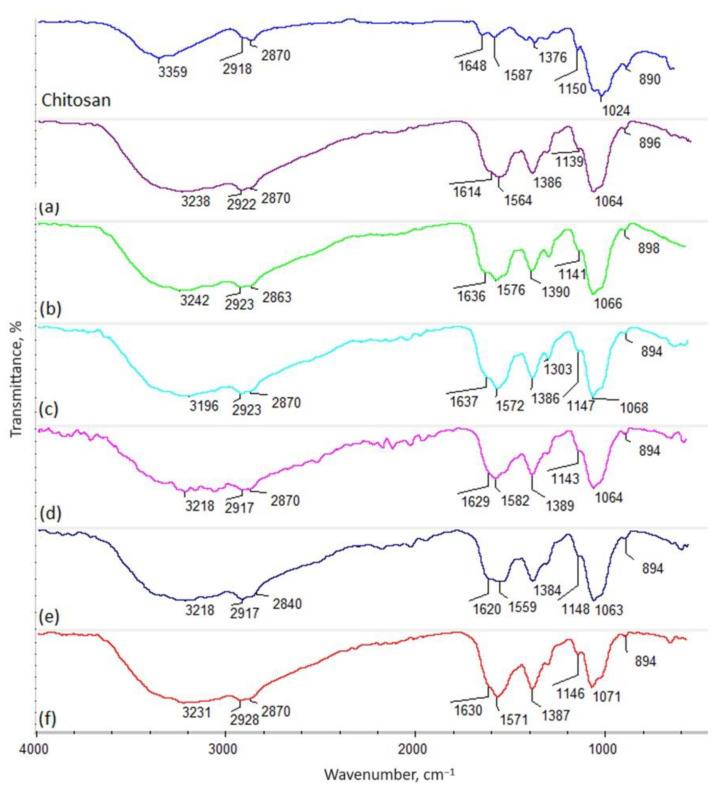
FT-IR spectrum: native chitosan—shrimp, 85% DD; (**a**) shrimp chitosan 85% DD crosslinked with aspartic and levulinic acid (frozen at −80 °C), sample a; (**b**) shrimp chitosan 95% DD crosslinked with aspartic and glutamic acid, sample b; (**c**) shrimp chitosan 90% DD crosslinked with glutamic acid modified with ZnO NPs, sample c; (**d**) shrimp chitosan 85% DD crosslinked with aspartic acid modified with Fe_3_O_4_, sample d; (**e**) shrimp chitosan 85% DD crosslinked with aspartic and levulinic acid, sample e; (**f**) squid chitosan crosslinked with aspartic acid, sample f.

Figure 2 presents the FT-IR spectra of the raw biopolymer (shrimp origin) and crosslinked biomaterials. The FT-IR spectra of the pure chitosan containing *N*-acetylaminoglucosamine and glucosamine units (Figure 2) reveal some bands typical of this polymer coming from hydroxyl groups at 3359 cm^−1^. Additionally, bands typical of aliphatic moieties are present at 2918 cm^−1^ (–CH_3_) and 2870 cm^−1^ (–CH_2_–). The spectrum shows amide bonds corresponding to *N*-acetyl mers at 1648 cm^−1^ of the chitosan polymeric chain. On the other hand, the presence of glucosamine mers can be proven by the bands at 1587 cm^−1^, as well as at 1150 cm^−1^, typical of free –NH_2_ groups. Additionally, bands corresponding to glycosidic bonds between mers are visible at 1024 cm^−1^. Finally, bands coming from glucopyranose rings can be spotted at 890 cm^−1^. Successful chemical modification via a microwave-assisted pathway using amino acids containing two different, reactive functional groups, such as carboxyl and amino ones, can be spotted for all investigated samples (b–f), as well as dicarboxyl acid (levulinic acid) (sample e). As the FT-IR spectra reveal, crosslinking occurs due to the formation of amide bonds between the –COOH coming from acids and the -NH_2_ present in glucosamine mers of chitosan. This phenomenon can be confirmed by a significant increase in the intensity of bands typical for OCN bonds around 1630 cm^−1^ (1614 cm^−1^ sample a; 1636 cm^−1^ sample b; 1637 cm^−1^ sample c; 1629 cm^−1^ sample d, 1620 cm^−1^ sample e; 1630 cm^−1^ sample f, respectively). Also, in each case, a shift of the band coming from free –OH groups in native chitosan to lower wavelengths can be seen due to the incorporation of free –COOH groups coming from amino acids and levulinic acid, as well as the destruction of the semi-crystalline regions present in unmodified chitosan, for which hydrogen bonds were responsible. Of note, no degradation occurred in any of the samples due to the exposure to microwave radiation, which is proven by the presence of bands coming from aliphatic moieties with unchanged intensities (around 2900 cm^−1^ and 2800 cm^−1^, respectively), as well as glycosidic bonds and glucopyranose rings (around 1020 cm^−1^ and 890 cm^−1^). Importantly, no decrease in the amount of the free amino groups can be observed (bands around 1560 cm^−1^ and 1150 cm^−1^). On the contrary, the choice of aspartic and glutamic acid enabled the incorporation of additional -NH_2_ moieties, which are known to be responsible for the favorable biological activities of the chitosan.

**Figure 3:**
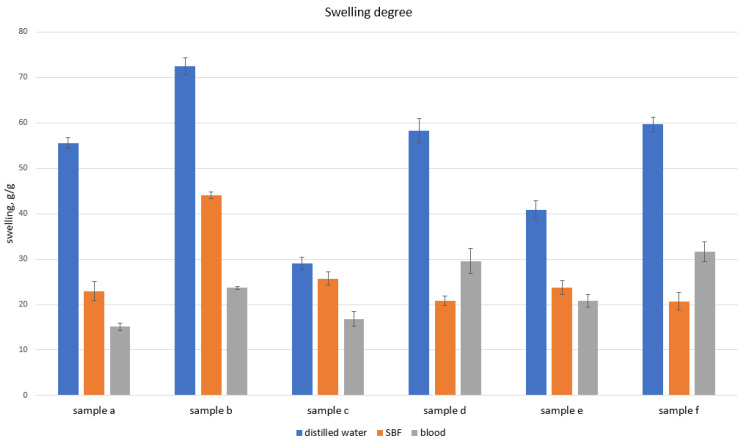
Swelling abilities in SBF and human blood.

The potential hemostatic activity was determined by evaluating the ability to absorb body fluids and human blood. The studies performed (Figure 3) show that all investigated samples became immediately swollen after immersing in the medium. Native chitosan, which is rich in free amino groups, is known for its hemostatic performance due to its electrostatic interaction with negatively charged erythrocytes. Chemical crosslinking resulting in the formation of highly porous structures rich in hydrophilic moieties provides the possibility to not only absorb high amounts of aqueous solutions, but also to create artificial clots. Moreover, the presence of NH_3_^+^, confirmed by FT-IR biomaterials, may positively affect the blood coagulation cascade under in vivo conditions.

### 2.2. Evaluation of Serum Levels of IL-6 and IL-10

Chitosan type B on day 7 significantly stimulated (*p* < 0.05) the increased secretion of IL-10 compared to IL-6, suggesting an anti-inflammatory effect. On day 14, both cytokines decreased, but IL-10 decreased significantly more, resulting in a tendency (*p* < 0.1) towards IL-6 dominance. By day 28, the proportions were equalized. This was the only procedure in which the average value of IL-10 was higher than that of IL-6 (not statistically significant, but the only one showing such a pattern).

Chitosan C on day 7 showed a tendency (*p* < 0.1) to stimulate pro-inflammatory activity (IL-6 dominance over IL-10). On days 14 and 28, IL-6 still was numerically larger, but was not statistically significant anymore.

After the injection on day 7, a slight IL-6 dominance was observed, followed by a reversal of this ratio in favor of IL-10 on day 14, and then a return to IL-6 dominance in the final observation on day 28 (*p* < 0.1). The injection had the least stable influence on IL-6 secretion.

**Figure 4:**
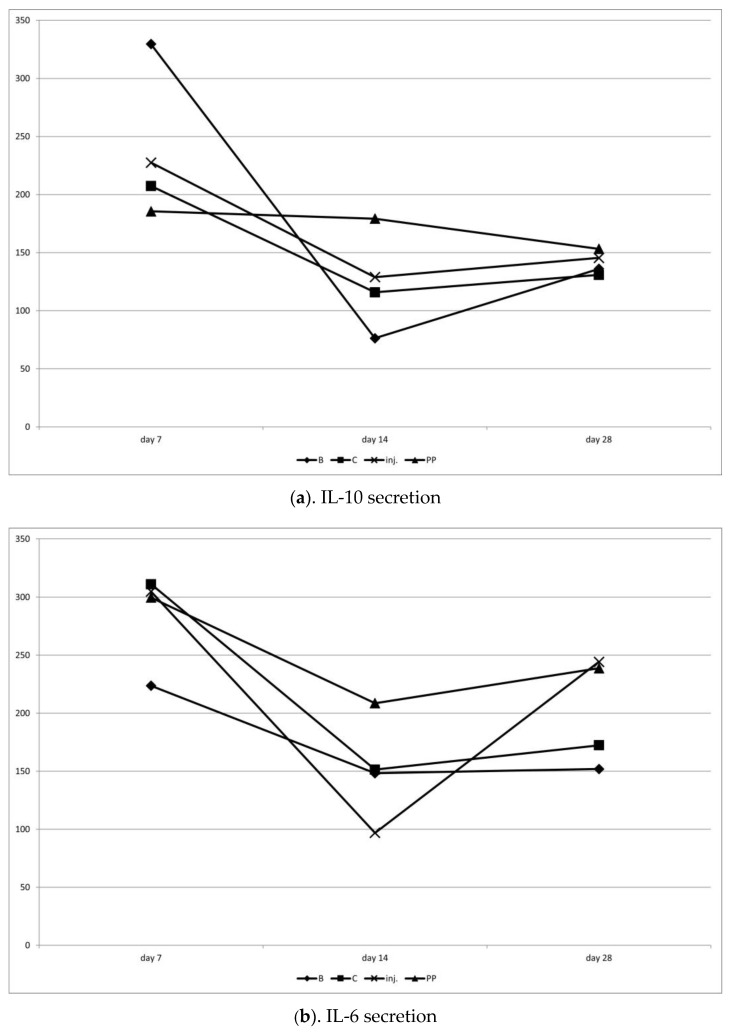

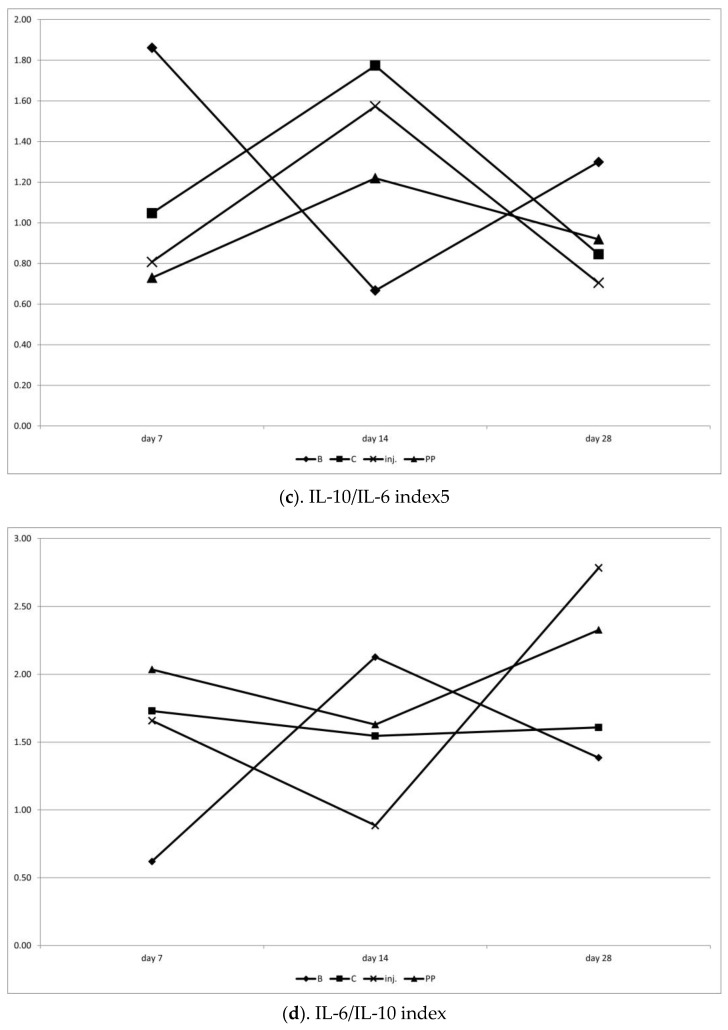
(**a**–**d**) Variation in IL-10 and IL-6 cytokine secretion, and indices of their mutual proportions on days 7, 14 and 28 after the surgery.

**Figure 5:**
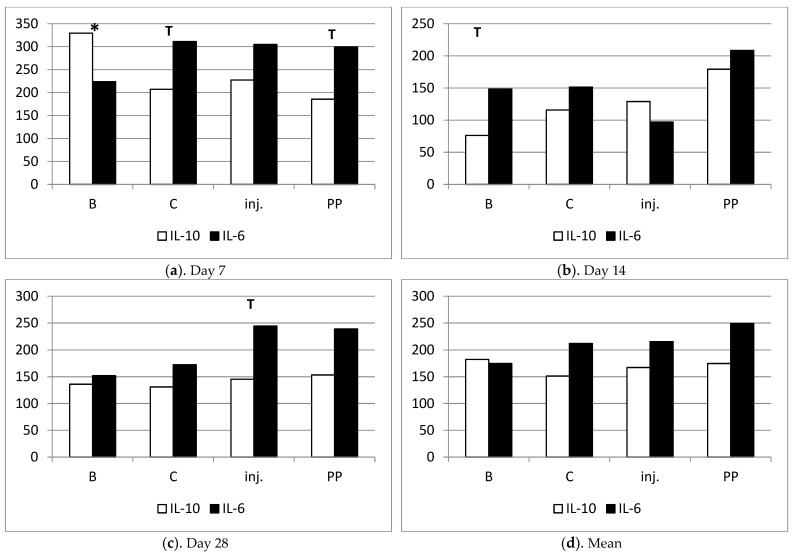
(**a**–**d**) Comparison of the secretion of anti-inflammatory (IL-10) and pro-inflammatory (IL-6) cytokines within the groups on individual days of observation. T—tendency to difference between values of IL-10 and IL-6 under symbol (*p* < 0.1); *—significant difference between values of IL-10 and IL-6 under symbol (*p* < 0.05).

Polypropylene showed the lowest and most stable secretion of IL-10 over time, with the highest and also most stable secretion of IL-6. This indicates the strongest pro-inflammatory influence of this procedure (Table 1; Figure 4 and Figure 5).

### 2.3. Pathomorphological Outcome

**Figure 6:**
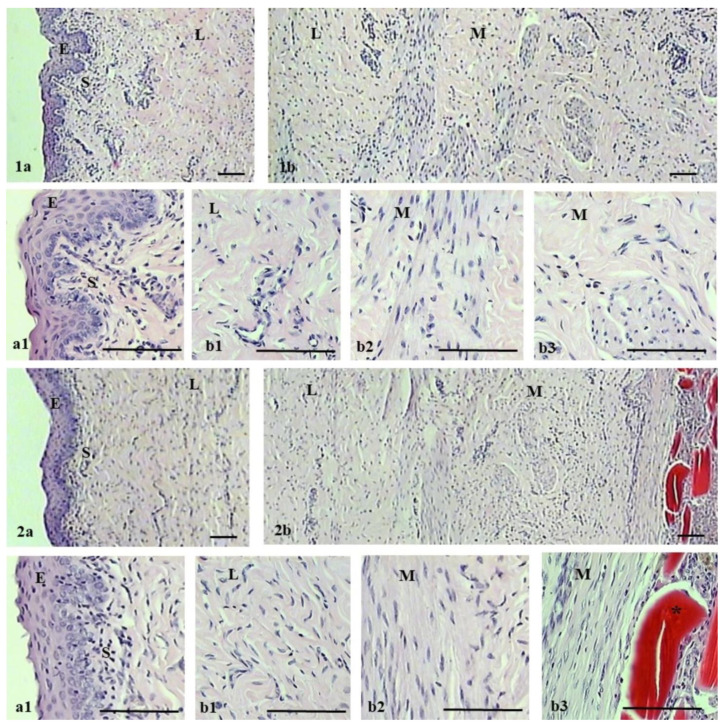
Hematoxylin–eosin staining of vagina in the control (**1a**,**1b**) and experimental (**2a**,**2b**) groups at 90 days after the surgery. For each group, the upper image shows vagina with epithelium on the left (**1a**,**2a**) and muscularis on the right (**1b**,**2b**). Lower images (**a1**,**b1**,**b2**,**b3**) show magnified portions (3×) of upper images (**1a**,**1b**,**2a**,**2b**): epithelium (E), subepithelium (S), lamina propria (L), and muscularis (M). Asterisk indicates areas with chitosan. Scale bar 100 µm.

The microscopic evaluation of the histological structure of the ovine vaginal wall showed a typical layered structure (Figure 6). The vaginal wall consists of four layers: non-keratinized stratified squamous epithelium, subepithelium or lamina propria of connective tissue, smooth muscle with bundles of circular and longitudinal fibers, and adventitia. The lamina propria was perforated by small arterioles and venules, and the muscularis consists of inner circular and outer longitudinal smooth muscle cells surrounded by connective tissue. In the case of the polypropylene group, histological observations showed no degenerative changes or inflammation. In contrast, chitosan injection in the experimental group caused multifocal accumulations of chitosan in the muscularis and, importantly, local inflammation in this area. No degenerative changes were observed in the other layers of the vaginal wall of the experimental group.

## 3. Discussion

The primary objective of our investigation was to elucidate the potential applications and biocompatibility of chitosan biomaterials in comparison to the commonplace polypropylene utilized in urogynecology. Leveraging the sheep model, we aimed to assess the material’s tolerance within the tissue environment and its impact on inflammatory parameters.

We employed a chitosan biomaterial, which lacks a predetermined structure characterized by biophysical durability. Notably, no adverse effects were observed within the chitosan group, unlike the polypropylene group, where we noted material extrusion into the vaginal lumen across all sheep.

Surgical interventions often elicit an inflammatory response encompassing the release of both pro- and anti-inflammatory interleukins [8]. Elevated levels of pro-inflammatory cytokine IL-6 and anti-inflammatory cytokine IL-10 in the blood serum of all assessed sheep on day 7 underscored the immune reaction following chitosan implementation. The surge in IL-6 levels on day 7 could also be attributed to tissue trauma during surgery. T cells and macrophages secrete IL-6, a potent pyrogen rapidly produced in response to tissue injury and infections, often preceding the elevation of body temperature and acute-phase protein release [9]. Serum IL-6 concentration mirrors the extent, duration, and nature of the surgical procedure.

IL-10, often termed cytokine synthesis inhibitory factor, dampens IL-6 and other proinflammatory cytokines [10]. Its concentration serves as an indicator of the surgical stress response [8]. Our study reveals a decline in IL-10 concentrations across all sheep after 14 days post-surgery, maintaining low levels throughout the study. Chitosan type B triggered a lesser proinflammatory IL-10 response compared to type C, while the injection variant displayed the most unstable IL-6 secretion. The polypropylene group triggered the highest inflammatory reaction, as evidenced by material extrusion into the vaginal lumen in all animals within this group. Given the rising prevalence of pelvic organ prolapse (POP) in women, the pursuit of a non-inflammatory response therapy underscores the promise of chitosan-based biomaterials in the future.

Chitosan scaffolds additionally exhibit the advantage of resembling the extracellular matrix (ECM) present in tissues. The porous structure facilitates cell proliferation, migration, and nutrient exchange. The controllable porosity of chitosan scaffolds proves beneficial for angiogenesis, a cornerstone supporting the viability and function of regenerated soft tissues [11,12]. These materials, in general, display non-toxic and biodegradable attributes, evoking minimal foreign body reactions with negligible fibrous encapsulation [13].

The obtained results show that the chemical modification of chitosan affects the inflammatory response of the host. Pristine chitosan, though, exhibits a resemblance to natural ECM components, especially glycosaminoglycans, which is generally known to cause a slight IL-6 increase after implementation, which, on the other hand, constitutes a common reaction after the injection of a foreign body. The evaluated chitosan scaffolds were modified chemically via a crosslinking process using two amino acids (*L*-aspartic and *L*-glutamic acids or pure *L*-glutamic acid alone), which are natural protein building blocks. Sample B (chitosan crosslinked with two amino acids) not only exhibited low inflammatory effects, but also had positive impacts on IL-10 secretion. Such an example of bioactivity can be assigned to the increased amount of free amine groups, which were introduced into the chitosan molecule by creating covalent, amide bonds, as shown in the FT-IR spectrum, which undergo protonation at higher pH values compared to native biopolymers. Of note, the chitosan free amino groups present in glucosamine mers protonate at pH values below 6.3, which may reduce their effectiveness. Sample C was modified with *L*-glutamic acid and ZnO NPs. The investigation into serum levels has shown that such a chemical composition induced a lower response from the immune system compared to chitosan and polypropylene mesh, yet did not induce anti-inflammatory effects in the same manner as sample B, which can be explained by the fact that ZnO nanoparticles more strongly affect the host organism, and are recognized as a foreign body.

A limitation of our study pertains to the relatively small sample size within the chitosan group. This size constraint may attenuate the predictability of multivariable regression modeling. Future endeavors should encompass the development of electrospinning techniques to generate chitosan mesh structures with the durability required for stabilizing pelvic organ prolapse.

## 4. Materials and Methods

### 4.1. Animals

The initial phase of our study encompassed the selection of an optimal animal model. While rodents (mice and rats) and rabbits are commonly utilized in biomedical research, often as preliminary steps with limited relevance to human medical applications, preclinical investigations for human health, diseases, and therapies predominantly center around porcine and ovine models.

Of these, pigs stand out due to their pronounced anatomical and physiological resemblances to humans, rendering them a favored choice. Notably, the resemblance between the pelvic floor and genital hiatus anatomy prompted researchers to focus on sheep as a prospective model for urogenital surgery [14]. Analogous similarities extend to the urogenital realm, where organ prolapse can manifest [15]. This ailment, linked with pregnancy and vaginal delivery (8 to 12% prevalence), can also be triggered by factors such as elevated intra-abdominal pressure (as in constipation), age, and frequency of multifetal pregnancy [16].

Although spontaneous pelvic organ prolapse is prevalent in sows, disparities in urogenital tract anatomy—such as a larger uterus and distinct vaginal-to-cervix transition—underscore the divergence between porcine and human mechanisms [17,18]. While sheep pelvis shape differs from that of humans and lacks certain structures like the sacrospinous ligament and internal obturator, certain components of the sheep pelvic floor mirror human pelvic anatomy. For instance, the levator ani muscle, though presented as a single entity, encompasses three separate muscles—the pubococcygeus, ileococcygeous, and coccygeus muscles—in the human pelvis [19].

Given their relatively modest cost and notable parallels in pelvic anatomy to humans, sheep emerge as a viable animal model in urogynecological investigations. For this study, 24 ewes of the Polish longwool sheep breed, aged between 2.5 and 3.5 years, were enlisted. They were randomly divided into four groups: one control and three experimental groups, each comprising 6 animals. All selected ewes had previously given birth.

The sheep were housed in group settings on deep bedding that was changed twice a year, within a conventional sheepfold featuring a functional attic. The sheepfold, affiliated with the Experimental Station of the Department of Animal Nutrition, Biotechnology, and Fisheries at the University of Agriculture in Kraków, maintained a minimum temperature of 8 °C and humidity levels below 70%. The facility upheld a natural light cycle, with two air exchanges per hour facilitated by natural ventilation. Each adult sheep enjoyed an average cage area of 1.5 m^2^.

During the period from May to October, the sheep were allowed pasture access during the day, while the rest of the year saw them housed in the sheepfold with access to a yard during feeding. The animals were tended to multiple times daily, receiving haylage and concentrates as per feeding standards, in addition to consistent water access. The sheepfold environment was enriched with straw and mineral licks. In preparation for the submucosal implantation procedure into the vaginal wall, the sheep underwent a 24-h fasting period, maintaining continuous water access.

### 4.2. Searching for a New Materials in Urogynaecology

The application of polypropylene meshes in urogynecological surgical procedures for organ prolapse repair has been linked to a range of adverse effects. These include the reduction in vaginal smooth muscle contractility and the degradation of structural collagen and elastin, leading to vaginal degeneration. Furthermore, the use of polypropylene meshes has been associated with the instigation of localized inflammatory responses. This underscores the imperative to explore alternative polymers suitable for mesh materials in urogynecological surgeries. The ideal candidate materials should possess sufficient tensile strength and facile integration with vaginal tissue, preserving the natural contractility of native tissue while evading foreign body responses and tissue fibrosis. Chitosan stands out as a particularly promising polymer, characterized by its exceptional attributes: biocompatibility, biodegradability, non-toxicity, limited immunogenicity, and antimicrobial properties [20].

### 4.3. Preparation for the Surgery, Proper Lining and Anaesthesia

The surgical procedure of chitosan injection was carried out under general anesthesia. The ewes did not receive food for 24 h prior to surgery but had unlimited access to water. An intramuscular tranquilizer Sedazin (2 mg/10 kg xylazine 2%, Biowet Puławy, Puławy, Poland) and—15 min later—intravenous anesthetic Ketamina (25 mg/10 kg ketamine hydrochloride, Biowet Puławy, Poland) were administered. After placing the ewe on the operating table, the vulva area was washed with soap and the skin was disinfected with ethanol (70%) and then iodine (3% iodine spirit solution in potassium iodide, Vetos-Farma Sp. z o.o., Bielawa, Poland); local submucosa anesthesia was performed by Polocaina (2 cc Polocainum Hydrochloricum 2% cum Adrenalino 0.005%, Biowet Drwalew, Drwalew, Poland) injection in the surgery area.

After the procedure of implanting a suitable chitosan or polypropylene implant and suturing (Amfil—P, Sinpo, Poznań, Poland), the ewes received Biovetalgin (metamizole sodium 500 mg/mL, Biowet Drwalew, Drwalew, Poland) and 2 mL of PenStrep antibiotic (procaine benzylpenicillin 200 mg/mL and Dihydrostreptomycin sulphate 250 mg/mL, Norbrook Laboratories Ltd. (Newry, UK), Scanvet Laboratories Ltd., Warsaw, Poland). After the procedure, the animals remained under veterinary care and received 2 mL of PenStrep antibiotic by intramuscular injection (musculus semimembranosus) for three consecutive days.

### 4.4. Surgery

**Figure 7:**
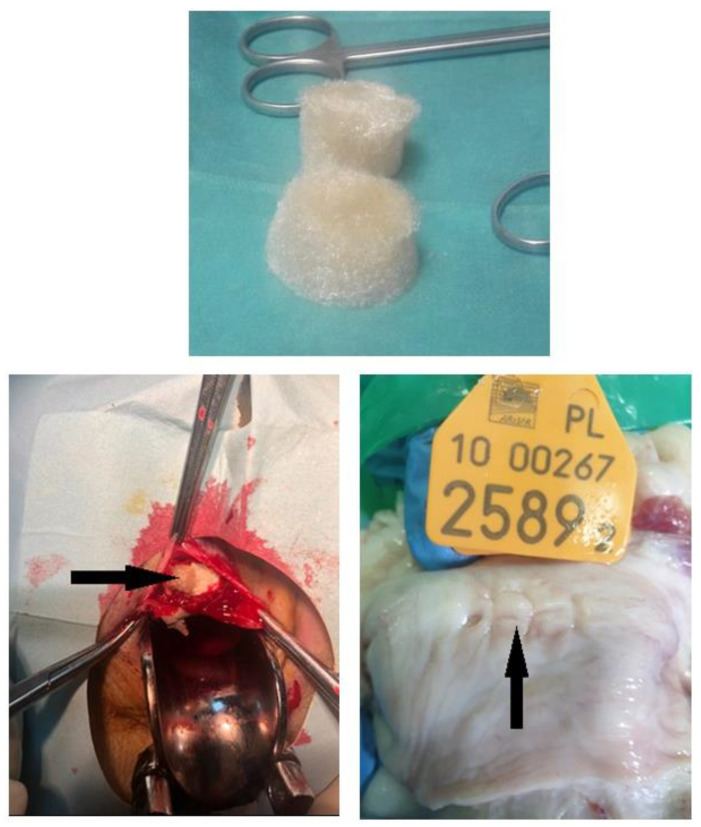
Chitosan pre-, intra-operatively and post-mortem with healed tissue.

Six chitosan type B and six chitosan type C samples were surgically implanted under the mucosa of the anterior vaginal wall. After the animal was put under the anesthesia, it was placed in the supine position with posterior legs bent to the body, with rectum emptied and vagina disinfected; the speculum was placed to visualize the anterior vaginal wall. Chitosan samples (type B or C in respective groups) were placed in the anterior vaginal wall under the mucosa at a depth of 3 mm, and the mucosa was closed with absorbable stiches. The next group comprised six chitosan unmodified injections performed 2 cm to 3 cm from under the external urethral os, to a depth of 3 mm. The injection volume was 4 mL. There was no bleeding from the injected area, no urine leakage, and no chitosan leakage. One suture was placed over the injection point to confirm the proper closing of the area. The other group featured the animals’ polypropylene mesh placed in six models, in the same way as with chitosan type B and C (Figure 7).

**Figure 8:**
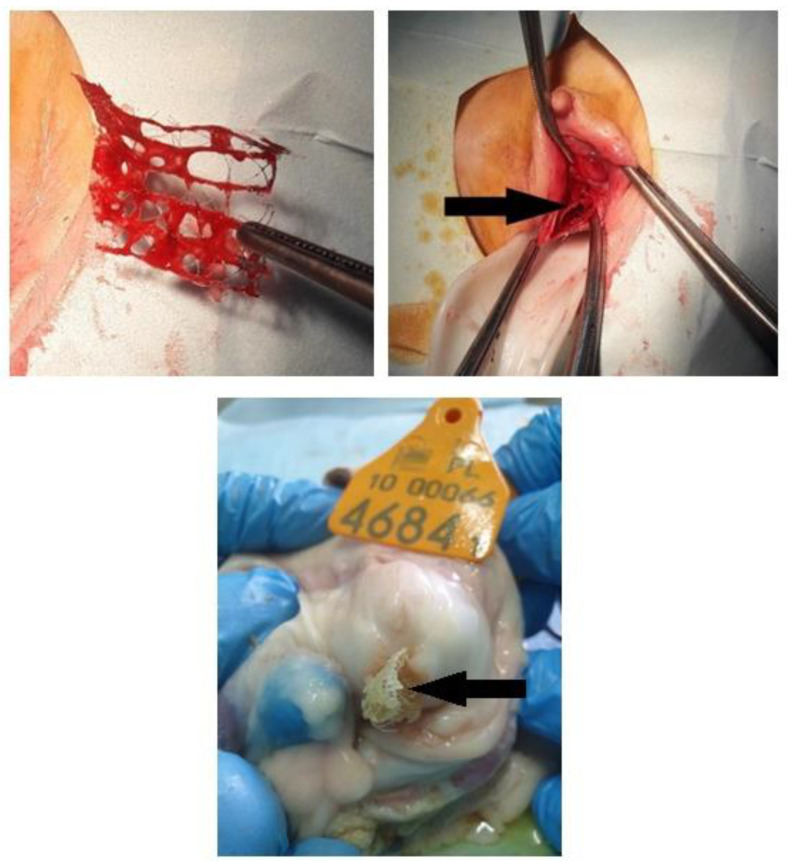
Polypropylene mesh pre- and intra-operatively and post-mortem with vaginal mesh extrusion.

Animals were sacrificed 90 days after the surgery. Post-implantation evaluation included macroscopical examination, and pathomorphological and immunohistochemical analyses (Figure 8).

### 4.5. Chitosan Biomaterials: Characterization

Tissue engineering is an important branch of science, focused on the repair and regeneration of damaged tissues. Designing material for tissue engineering is a challenge for regenerative medicine.

Three different types of chitosan biomaterials (A, B and C, respectively, according to the animal groups) were used for the study. Type A was unmodified chitosan injection of marine origin (85% deacetylation) dissolved in lactic acid. The microwave-assisted synthesis of novel chemically crosslinked scaffolds with hemostatic properties (types A–F) was carried out using a domestic oven. After the reaction, the samples were washed out with distilled water to neutral pH, frozen at −80 °C (type A) or −20 °C (types B–F), and freeze-dried. Types C and D used in our animal sheep model were additionally functionalized with ZnO and Fe_3_O_4_ nanoparticles, respectively. For this purpose, dry nanoparticles were added to the reaction mixture before placing in the microwave reactor and treated with ultrasounds to prevent agglomeration. Zinc oxide and iron (II, III) oxide nanoparticles (ZnO, Fe_3_O_4_ NPs) were obtained using the methods described in our previous paper [21] (Table 2).

The nanoparticles were characterized via their size and shape using TEM (transmission electron microscopy). The chemical structure of the crosslinked chitosan biomaterials was investigated with Fourier-Transform Infrared Spectroscopy using a Thermo Nicolet Nexus 470 FT-IR spectrometer equipped with an ATR adapter. The samples were tested for their hemostatic and swelling abilities with distilled water, simulated body fluid (SBF), and rehydrated blood (Sigma Aldrich, St. Louis, MO, USA). Swelling degree was calculated using following equation:

SD = W_t_ − W_0_/W_t_ × 100%,

where 

SD—swelling degree, %,

W_t_—weight after immersing in SBF/blood for 5 min and

W_0_—weight of a dry sample.

Also, their porosity was verified with the isopropanol displacement method.

### 4.6. ELISA Assay: IL-6, IL-10

Blood samples were collected from 24 sheep at three time points: the 7th, 14th and 28th day after implantation of the material, respectively. The samples were centrifuged, and the collected serum was used in an ELISA assay for the quantitative detection of IL-6 and IL-10 (FineTest, Wuhan Fine Biotech Co., Ltd., Wuhan, China). The tests were performed following the manufacturer’s protocols and analyzed on the Spark Multimode Microplate Reader 10 M (TECAN, Männedorf, Switzerland) at 450 nm. All samples were assayed in duplicate. Concentrations of cytokines for each sample were calculated based on optical density (OD) readout and expressed in pg/mL.

### 4.7. Pathomorphology

The sections from the vagina were fixed in 10% buffered formaldehyde (pH 7.0) for 24 h. The formaldehyde-fixed samples were embedded in paraffin in a routine procedure. The samples were cut with a microtome into 6 μm sections (Leica RM2146 microtome, Nußloch, Germany) and stained with hematoxylin and eosin (H&E) (Sigma-Aldrich, Darmstadt, Germany) for morphological examination using a light microscope (NIKON E600, Tokyo, Japan).

### 4.8. Statistical Analyses of Data

Analyses of data in terms of cytokine levels were performed with the Statistica 13 software (Statsoft Poland, Kraków, Poland) to determine the main inflammatory effects of the procedure performed during the surgery. The data were initially subjected to a distribution test (Shapiro–Wilk test) and screened for potential outliers using Grubb’s test. Subsequently, comparisons among groups were performed using ANOVA followed by Duncan’s multiple range test for individual IL-6 and IL-10 levels and their changes in time after surgery in every group. Additionally, the proportions of IL-6 to IL-10, and of IL-10 to IL-6, were calculated as the index value to show cumulative pro-inflammatory or anti-inflammatory effects. These data were also analyzed to compare the effects of procedures among groups, using ANOVA followed by Duncan’s test. The data are presented as mean ± SEM.

## Figures and Tables

**Table 1:** Mean values of IL-10 and IL-6 cytokine secretion and indices of their mutual proportions throughout the study.

Item	IL-10	IL-6	IL-10/IL6	IL-6/IL-10
Chitosan B	182.28 ± 32.36	174.65 ± 13.53	1.23 ± 0.20	0.88 ± 0.14
Chitosan C	151.35 ±16.19	211.64 ± 34.02	0.99 ± 0.40	1.53 ± 0.29
Chitosan injection	167.29 ± 32.69	215.64 ± 28.48	0.86 ± 0.23	1.60 ± 0.34
Polypropylene	174.80 ± 36.40	248.99 ± 49.72	0.93 ± 0.36	1.98 ± 0.69

**Table 2:** Preparation of chitosan samples.

Type/Group	Chitosan Type, g	Crosslinking Agent, g	High Boiling Solvent, mL	Nanoadditive, mg	Power, W	Time, min
A	Shrimp, 85% DD, −80 °C freeze	Levulinic acid, aspartic acid; 0.5:0.5	Propylene glycol, 10	---	800	3
B *	Shrimp, 95% DD	Aspartic acid, glutamic acid; 0.5:0.5		---		
C *	Shrimp, 90% DD	Glutamic acid; 0.84		ZnO NPs, 8		
D	Shrimp, 85% DD	Aspartic acid, 0.84		Fe_3_O_4_ NPs, 8		
E	Shrimp, 85% DD	Levulinic acid, aspartic acid; 0.5:0.5		---		
F	Squid, 85% DD	Aspartic acid, 0.84		---		

* Based on our preliminary in vitro study results, groups B and C were selected for in vivo experiments, as they were the most promising since they exhibited no cytotoxicity.

## Data Availability

The authors declare that all the data supporting the findings of this study are provided within the article.
